# Effects of One-Week Empirical Antibiotic Therapy on the Early Development of Gut Microbiota and Metabolites in Preterm Infants

**DOI:** 10.1038/s41598-017-08530-9

**Published:** 2017-08-14

**Authors:** Danping Zhu, Sa Xiao, Jialin Yu, Qing Ai, Yu He, Chen Cheng, Yunhui Zhang, Yun Pan

**Affiliations:** 1 Department of Neonatology, Children’s Hospital of Chongqing Medical University, Chongqing, China; 2 Ministry of Education Key Laboratory of Child Development and Disorders, Chongqing, China; 3 China International Science and Technology Cooperation Base of Child development and Critical Disorders, Chongqing, China; 4 Chongqing Key Laboratory of Child Infection and Immunity, Chongqing, China; 5 Department of Pediatric, The Affiliated Hospital of Shenzhen University, Shenzhen, China

## Abstract

The early postnatal period is the most dynamic and vulnerable stage in the assembly of intestinal microbiota. Antibiotics are commonly prescribed to newborn preterm babies and are frequently used for a prolonged duration in China. We hypothesized that the prolonged antibiotic therapy would affect the early development of intestinal microbiota and their metabolites. To test this hypothesis, we analyzed the stool microbiota and metabolites in 36 preterm babies with or without antibiotic treatment. These babies were divided into three groups, including two groups treated with the combination of penicillin and moxalactam or piperacillin-tazobactam for 7 days, and the other group was free of antibiotics. Compared to the antibiotic-free group, both antibiotic-treated groups had distinct gut microbial communities and metabolites, including a reduction of bacterial diversity and an enrichment of harmful bacteria such as *Streptococcus* and *Pseudomonas*. In addition, there was a significant difference in the composition of gut microbiota and their metabolites between the two antibiotic-treated groups, where the piperacillin-tazobactam treatment group showed an overgrowth of *Enterococcus*. These findings suggest that prolonged antibiotic therapy affects the early development of gut microbiota in preterm infants, which should be considered when prescribing antibiotics for this population.

## Introduction

With an estimated average of 10^14^ bacterial cells and over 400 species residing in an adult^1^, the gut microbial community is indispensable for human health. Compared to adults, the total number of gut bacterial species is much lower in neonates, especially in preterm infants (with a gestational age between 28 and 37 weeks)^2, 3^. However, the neonatal microbial dynamics exerts an extensive and far-reaching influence on the microbiota assembly. It has been reported that the neonatal microbiota disturbance may lead to obesity, asthma and other allergic diseases^4^, whereas a balanced, healthy microbiota can protect infants from many diseases. The gut microbiota is highly variable during early infancy and is influenced not only by the gestational age, delivery mode, and feeding method, but also by the medical treatment. Compared with term infants (with a gestation of 37–42 weeks), preterm neonates have a distinct intestinal microbiota, with a delayed colonization of common bacteria, such as *Bifidobacteria* ^2^, and a higher prevalence of pathogens, such as *Clostridia* ^5^. The abnormal gut microbiota in preterms may be due to the premature birth itself and other factors, including exposure to the hospital environment after birth or medical interventions, particularly the use of antibiotics^6, 7^.

Considering that preterm babies are prone to infections due to their immature immune system and that current diagnostic tests for neonatal sepsis have poor positive predictive values, clinicians often prescribe antibiotics to them shortly after birth, especially after the onset of chorioamnionitis in mothers and premature rupture of membranes. While in general antibiotics should be discontinued after 48 hours if the laboratory tests indicate a low probability of sepsis^8^, prolonged use of antibiotics is very common due to various concerns. Nowadays, increasing attention has been paid to the adverse effects of prolonged antimicrobial therapy. There have been reports of association of prolonged initial empirical antibiotic treatment with late onset sepsis (LOS), necrotizing enterocolitis (NEC), and death in preterm infants^9, 10^. More recently, it has been reported that empiric antibiotic therapy can result in a lower bacterial diversity and a higher abundance of *Enterobacter* in the guts of newborns (with a gestation ≤32 weeks)^11^ and term infants^12^ during their first month. However, the effects of antibiotic use on the gut microbiota and metabolites during the first week after birth remain undefined. The first week of the postnatal period is the most dynamic and vital stage in the establishment of intestinal microbiota. Several studies suggested that a decrease in the microbiota diversity in the first seven postnatal days may lead to asthma and eczema during childhood^13–15^. It is unknown how prolonged use of antibiotics affect the gut microbiota during this period.

Among the most commonly prescribed antibiotics to preterm babies are the broad-spectrum β-lactam antibiotics, which contain a β-lactam ring in their molecular structures. This class of antibiotics includes penicillin and its derivatives (such as piperacillin, monobactams, cephalosporins, and carbapenems). Most β-lactam antibiotics act by impeding the formation of bacterial cell wall. Since bacteria often develop resistance to β-lactam antibiotics by synthesizing β-lactamase, an enzyme that degrades the β-lactam ring, they are usually given in combination with β-lactamase inhibitors (such as tazobactam)^16^. Moxalactam is an oxa-β-lactam antibiotic that inactivates β-lactamase and also is a broad spectrum antibiotic that works against Gram-positive and Gram-negative anaerobic and aerobic bacteria, especially Gram-negative bacillus, such as *Enterobacteriaceae* ^17^.

In our study, the combination of the classical β-lactam antibiotic penicillin and the new synthetic β-lactam antibiotic moxalactam (penicillin-moxalactam), and the combination of the extended-spectrum penicillin-derivative piperacillin and the β-lactamase inhibitor tazobactam (piperacillin-tazobactam). The primary goal of this study was to explore the effects of one-week antibacterial treatment on the gut bacterial community and their metabolites in preterm infants during the first week after birth. In addition, we examined the differences in gut bacterial community and their metabolites between preterm infants receiving different antibiotics, including the combination of penicillin-moxalactam (PM group) and piperacillin-tazobactam (PT group).

## Results

### Demographic and Clinical Characteristics of Enrolled Patients

Demographic and clinical background information of the enrolled preterm infants is shown in Table 1. The subjects of the three groups, including two treatment groups (PM and PT groups) and antibiotic-free group (AF group), were well case matched. There were no statistically significant differences in any of the demographic and clinical characteristics compared among these three groups. All preterms were formula-fed and none of them received supplemental probiotics or prebiotics during the study period.

**Table 1:** Demographic and clinical characteristics of study subjects.

	AF group (n = 12)	PT group (n = 12)	PM group (n = 12)	P value
Newborns’ characteristics				
Gestational age, mean (week)	34.4	34.3	34.3	0.987
Birth weight, mean (g)	2,250	2,162	2,027	0.438
APGAR 5 min score, median	10	10	10	1.0
Male/Female	4/8	4/8	6/6	0.629
Vaginal birth, n (%)	2 (17)	2 (17)	2 (17)	1.0
Twin birth, n (%)	2 (33)*	3 (50)*	2 (33)*	0.842
Mothers’ conditions				
Rupture of membranes (>18 h), n (%)	1 (8)	5 (42)	5 (42)	0.089
Hypertension/pre-eclampsia, n (%)	5 (42)	1 (8)	2 (17)	0.126

*Two subjects were counted for each twin in calculating the percentages. Fisher’s Exact test was used for categorical variables and Kruskal-Wallis Test was used for continuous variables. P < 0.05 was considered as significant.

### Diversity Analysis of Microbiota

**Figure 1:**
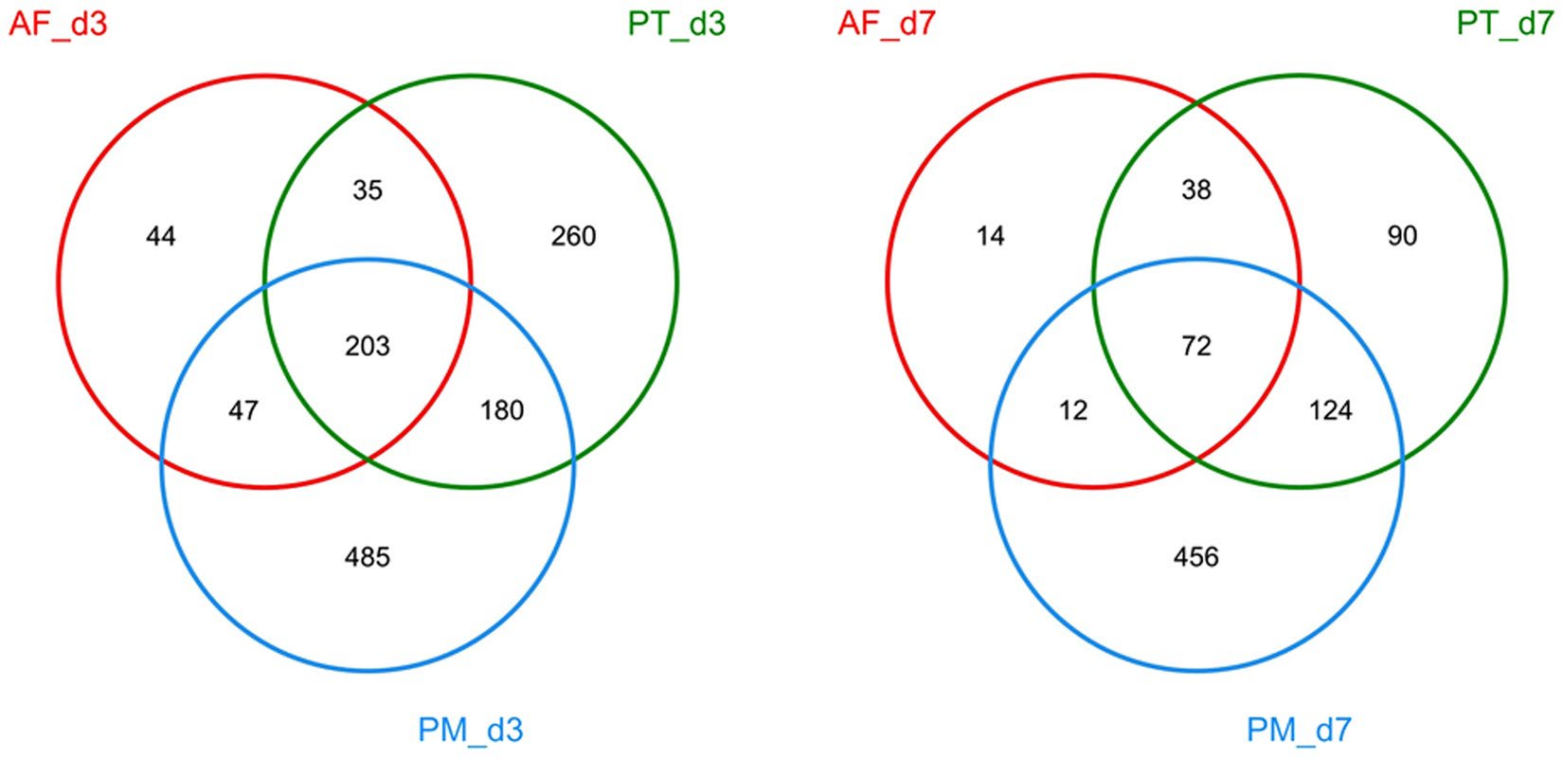
Venn diagrams of the distribution of Operational Taxonomic Units (OTUs) among three study groups on day 3 (d3) and day 7 (d7). The number in the intersection represent the number of OTUs shared among different groups while the number out of the intersection represents the number of unique OTUs in each group. AF, antibiotic free group (red color); PT, piperacillin-tazobactam group (green color); PM, combination of penicillin and moxalactam group (blue color).

Fecal samples collected from all enrolled preterms were successfully amplified by the universal bacterial 16 S rRNA primers except for the meconium samples from all preterms and one fecal sample (on day 3) from one preterm in the AF group. High-throughput sequencing of the positive PCR products by the Illumina MiSeq platform generated a total of 1,145,656 valid sequences (passing quality control) with an average of 16,136 sequences per sample (range 10,084–28,177). The number of bacterial operational taxonomic units (OTUs) in each sample ranged from 5 to 373. The OTUs among the three study groups were compared via Venn diagrams, which showed that 203 and 72 OTUs were shared among each group on day 3 (d3) and day 7 (d7), respectively (Fig. 1). These shared bacterial OTUs accounted for a minor proportion of total bacterial community in both the PT and PM groups but dominated in each of the AF group. The number of shared OTUs decreased on d7 compared to d3.

**Figure 2:**
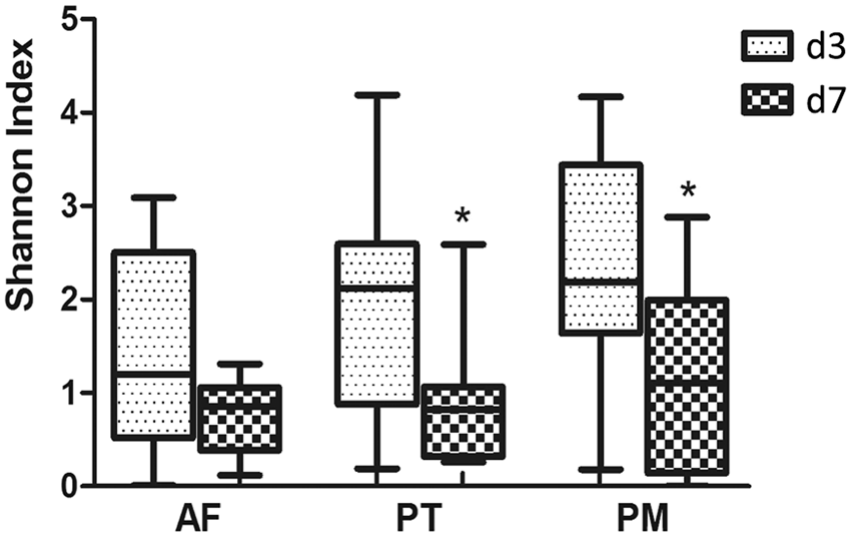
Comparison of gut microbial diversity among PT and PM treatment groups and AF control group on d3 and d7. A bigger Shannon index indicates a higher microbial diversity. The inside bar represents the median, the outer horizontal line of the box represents the 25th and 75th percentiles. Error bars represent the standard error. *Indicates significant differences in the Shannon index between PT and AF groups (P = 0.028) and between PM and AF groups (P = 0.008). For the abbreviation of group names, see Fig. 1 legend.

The Shannon index, which was calculated based on the number and distribution of OTUs, showed no statistical difference among the three groups on both d3 and d7 (Fig. 2). Nevertheless, both PM and PT groups showed significantly lower Shannon index on d7 compared to those on d3 (P = 0.028 for PT, P = 0.008 for PM). There was no significant difference in the Shannon diversity index between the PM and PT groups at either time point.

**Figure 3:**
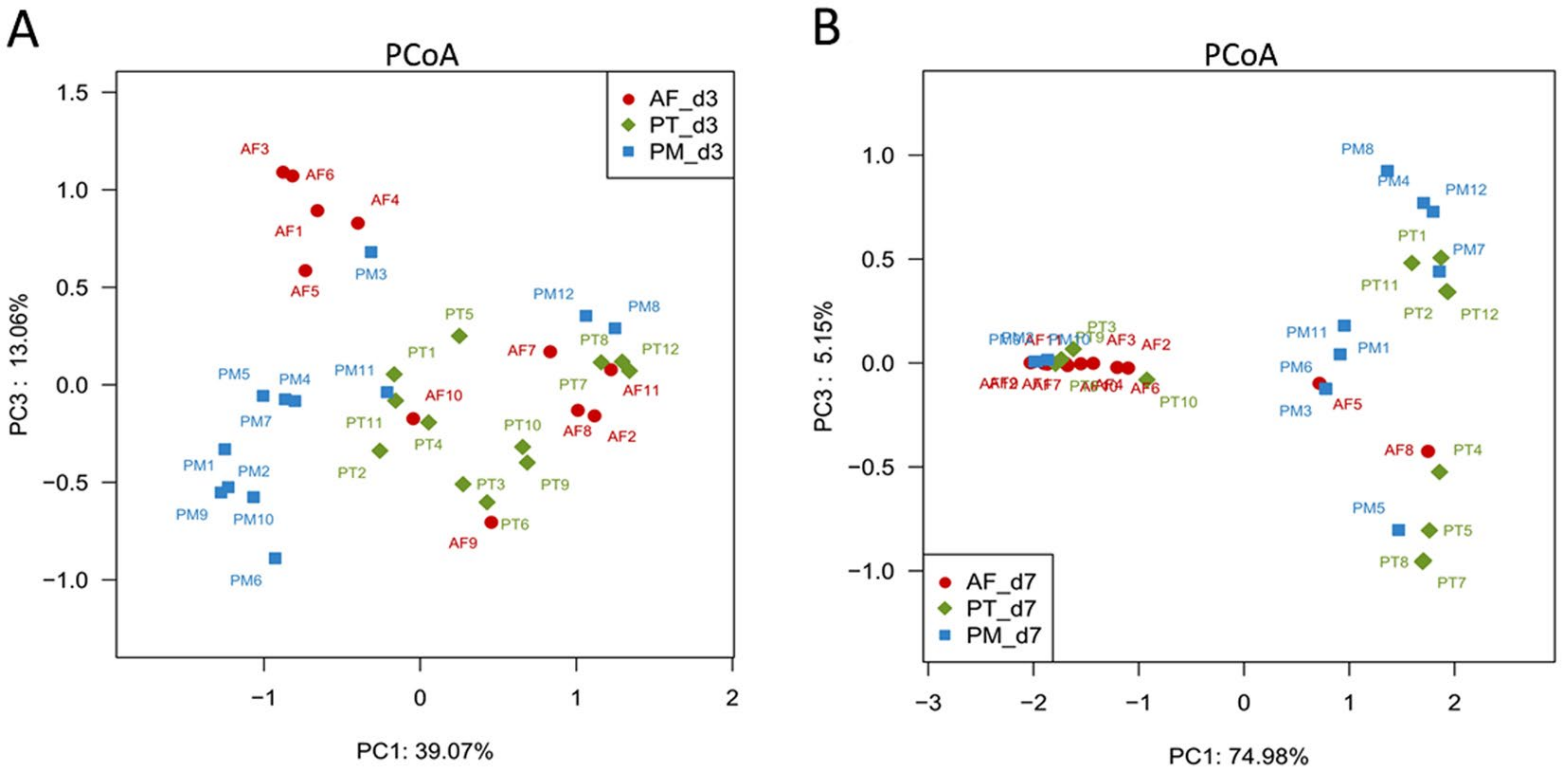
Principal coordinate analysis (PCoA) of microbial communities among PT and PM treatment groups and AF control group. (**A**) Distribution of all samples on d3; (**B**) Distribution of all samples on d7. Samples that are positioned close to each other are similar to each other in microbial compositions whereas samples that are positioned distantly from each other are distinct to each other in microbial compositions. AF, antibiotic free group; PT, piperacillin-tazobactam group; PM, combination of penicillin and moxalactam group.

Principal coordinates analysis (PCoA) showed a significant difference in bacterial compositions among the three groups. All the subjects were well separated from each other on d3, while 10 out of the 12 AF subjects clustered together on d7. The subjects of the two antibiotic treatment groups were quite distant from each other, based on the first two principal component scores, which accounted for 75% and 5% of the total variations, respectively (Fig. 3).

### Composition Analysis of Microbiota

**Figure 4:**
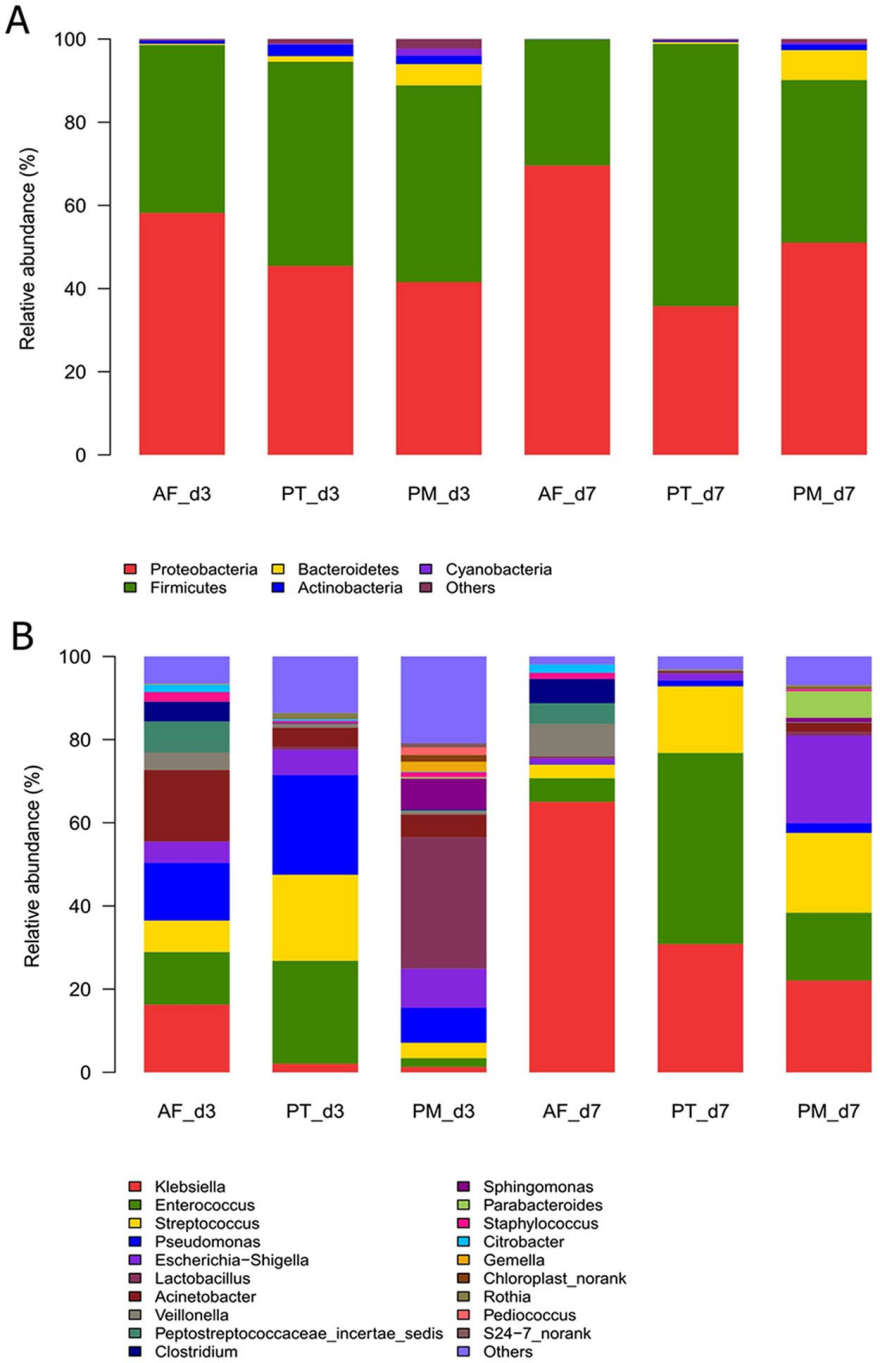
Comparison of the relative abundance of bacterial communities among PT and PM treatment groups and AF group on d3 and d7. (**A**) Relative abundance at the phylum level. (**B**) Relative abundance at the genus level. Different phyla and genera are color-coded. AF, antibiotic free group; PT, piperacillin-tazobactam group; PM, combination of penicillin and moxalactam group.

The overall microbiota composition of each group at the phylum and genus levels is shown in Fig. 4. At the phylum level, Firmicutes and Proteobacteria were the most abundant in all enrolled subjects on d3 and remained dominant on d7. Bacteroidetes and Clostridia were rarely detected. In both AF and PM groups, there was an approximately 10% increase in the proportion of Proteobacteria, whereas Firmicutes decreased by the same percentage on d7 compared to d3 during the first week. In contrast, the proportions of these two major bacterial phyla in the PT groups showed an opposite trend. At the genus level, the distribution of bacteria in the AF group was uniform on d3. *Enterococcus*, *Streptococcus*, and *Pseudomonas* accounted for more than 60% of the microbiota in the PT group on d3. The proportion of *Lactobacillus* was significantly higher in PM group (31.57%) than the other two groups on d3 (P < 0.001). Also, the proportion of *Klebsiella* increased among all three groups on d7 compared to d3 and it dominated in the AF group on d7 (65%). The top two genus in the PT group on d7 were *Enterococcus* and *Klebsiella*, which accounted for 46% and 31%, respectively. *Enterococcus*, *Streptococcus*, *Klebsiella* and *Enterobacter* were frequently detected in the PM group. The bacterial composition of all enrolled samples is shown in detail in Supplementary Fig. S1, where the heatmap exhibits the distribution of the top 100 abundant genera among all samples.

A cladogram representation of the composition of fecal microbiota and their predominant bacteria is shown in Supplementary Fig. S2. Significant variations in the composition of fecal microbiota were observed among the three groups. Detailed analysis of the phylum and genus levels on d3 (Fig. 4) showed no difference in the distribution of the two predominant phyla (Proteobacteria and Firmicutes) among the three groups, while Bacteroidetes and Actinobacteria were significantly more abundant in the PM and PT groups compared to the AF group, respectively. At the genus level, *Sphingomonas*, *Bacteroides*, and *Lactobacillus* were more abundant while *Clostridium* was less abundant in the PM group compared to the AF group (P < 0.05). There was no significant difference in the bacterial composition between the AF and PT groups. Nevertheless, *Enterococcus* was more prevalent in the PT group than in the PM group (P = 0.003).

We also assessed the changes in the fecal microbiota on d7 (Fig. 4). At the phylum level, the distribution of Firmicutes was distinct among the three groups, with the proportion of *Bacteroidetes* being higher in the two antibiotic treatment groups than in the AF group (P < 0.05). At the genus level, several genera exhibited a significant difference in their relative abundance among the three groups. Compared with the AF group, the PT group but not the PM group showed a higher prevalence of *Enterococcus* and lower prevalence of *Klebsiella* (P = 0.003 for *Enterococcus*, P = 0.028 for *Klebsiella*). *Escherichia-Shigella* was more prevalent in the PM group but not the PT group compared to the AF group (P = 0.018). The comparison between the PT and PM group revealed a higher prevalence of *Enterococcus* and a lower prevalence of *Clostridium* in the PT group (P = 0.004 for *Enterococcus*, P = 0.005 for *Clostridium*).

### Fecal Metabolomic Analysis

**Figure 5:**
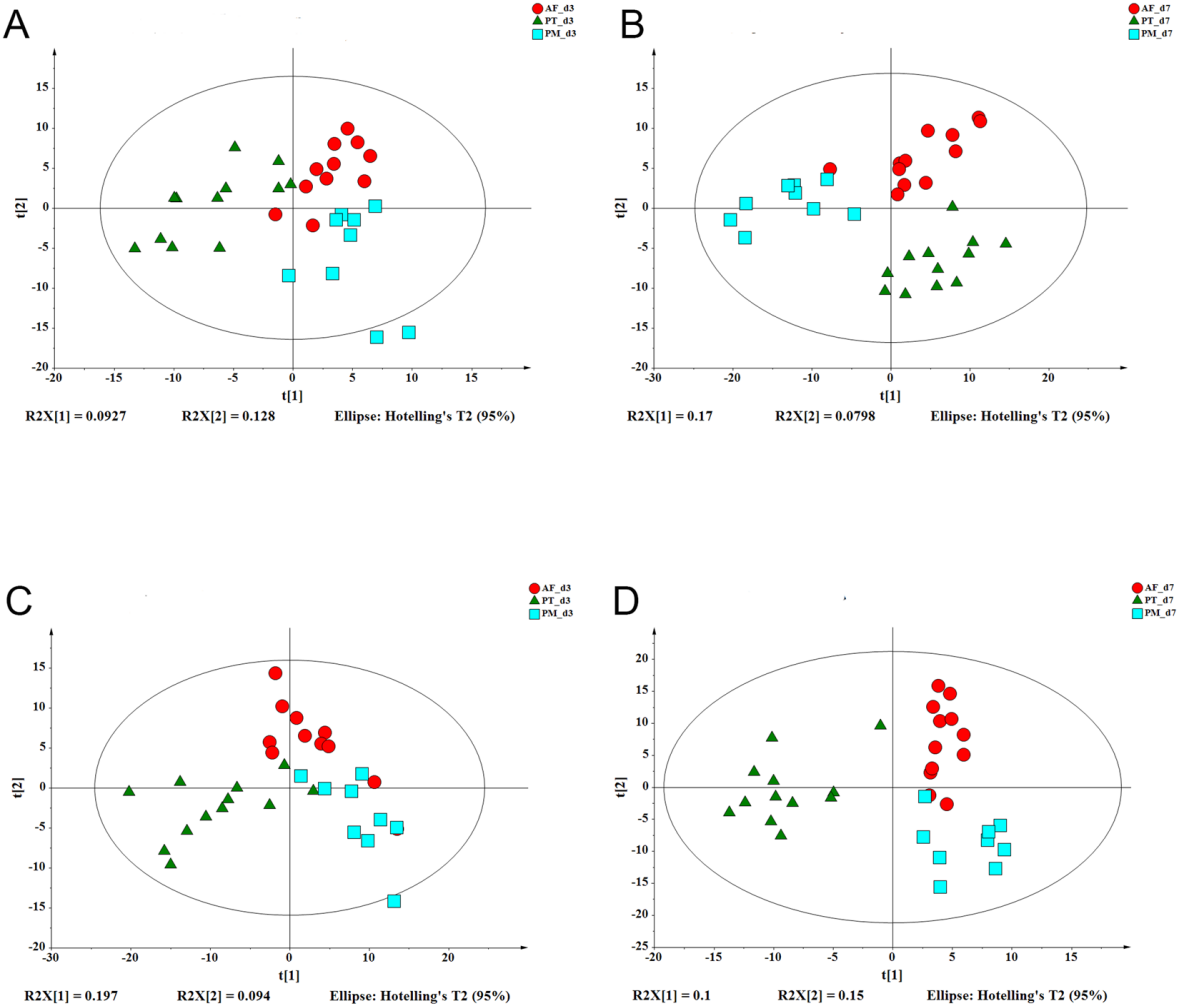
Partial least squares-discriminant analysis (PLS-DA) score plots of metabolite profiles on d3 and d7. (**A** and **B**) Negative ion mode. (**C** and **D**) Positive ion mode. The ellipse area represents the 95% confidence interval by Hotelling T2 test. The red circle represents each sample in the antibiotic free group; the green triangle represents each sample in the piperacillin-tazobactam group; the blue square represents each sample in the combination of penicillin and moxalactam group.

Based on supervised partial least squares-discriminate analysis (PLS-DA) model, the differences in metabolite compositions among the three groups were more remarkable on d7 than on d3. The PLS-DA score plots are shown in Fig. 5. A total of 37 kinds of metabolites were identified by metabolomic analysis. The correlation analysis of fecal metabolites and bacterial compositions is shown in Supplementary Figs S3A and B and S4A and B. At the phylum level, the proportion of Bacteroidetes in the PM group and the proportion of Actinobacteria in the PT group were the highest on d3. Bacteroidetes was positively correlated with L-valine and L-serine, both of which were mostly detected in the PM group. Glycerol and L-proline were dominant in the PT group and positively correlated with Actinobacteria, which was also dominant in the PT group on d3. Although gluconic acid and Proteobacteria were most abundant and showed a positive correlation in the AF group, the difference in the proportion of Proteobacteria was not statistically significant among the three groups on d3.

On day 7, the distribution of dominant fecal bacteria in the AF group remained the same as on d3. Only four metabolites (linoleic acid, L-glutamate, L-valine, and pantothenic acid) significantly increased on d7 compared to d3 (P < 0.05) and were all positively correlated with Proteobacteria, which were the most abundant phylum in the AF group. In addition, three of these four metabolites (L-glutamate, L-valine, and pantothenic acid) were negatively correlated with the Firmicutes, Actinobacteria and Bacteroidetes, which were abundant in both antibiotic treatment groups. The PM group had the highest abundance of L-tyrosine and citric acid among the three groups, where citric acid was positively correlated with the abundant bacteria in the antibiotic treatment groups and was negatively correlated with the dominant bacteria in the AF group. At the genus level, *Sphingomonas*, *Lactobacillus*, *Bacteroides* were mostly present in the PM group while *Enterococcus*, *Pseudomonas* were mostly present in the PT group. Compared with the PM and PT groups, only *Clostridium* was the most enriched in the AF group.

The significant differences in metabolites among the three groups at the genus level was the same as the phylum level, but the correlation analysis of the metabolites and bacteria showed no apparent tendency at the genus level on d3. Nonetheless, opportunistic pathogens *Enterococcus*, *Pseudomonas* and pathogenic *Escherichia-Shigella* were more prevalent in antibiotic treatment groups than in the AF group (P < 0.05) on d7, and were positively correlated with L-tyrosine and citric acid, which were significantly different in their distribution among the three groups (P < 0.05). The AF group had the highest proportion of *Peptostreptococcus*, *Klebsiella*, and *Clostridium*, which were positively correlated with L-valine, L-glutamate, pantothenic acid and linoleic acid. In addition, almost all bacteria that are abundant in the antibiotic treatment groups were negatively correlated with these four metabolites.

**Figure 6:**
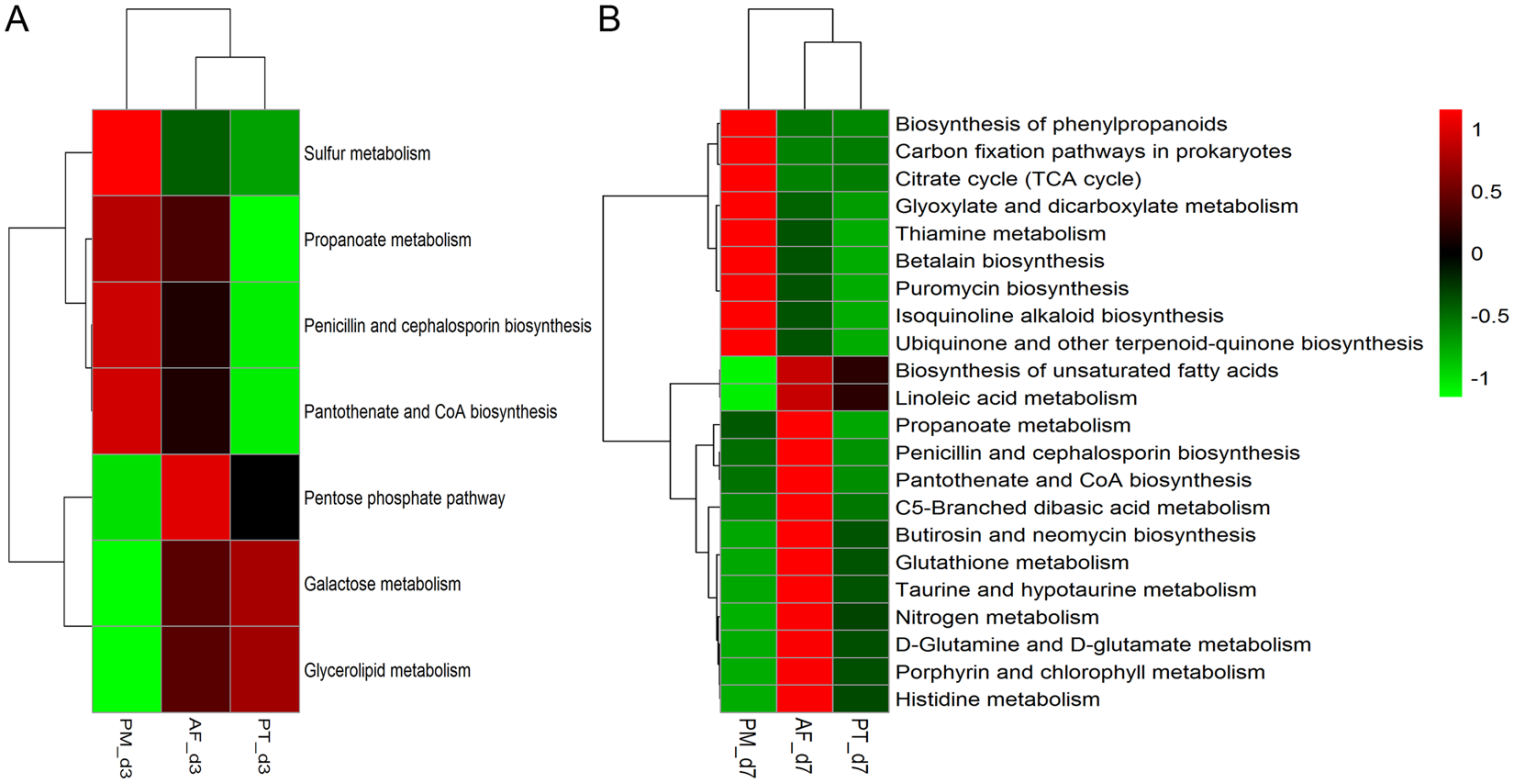
Heatmap of significantly different metabolic pathways of the three study groups on d3 (**A**) and d7 (**B**). One-way ANOVA test was used for the comparison of metabolic pathways among the three groups (P < 0.05). The cluster analysis was based on the processes of metabolism. The activity score (AS) calculated by Pathway Activity Profiling (PAPi) ranged from −1 to 1. For the abbreviation of group names, see Fig. 1 legend.

All identified metabolites were mapped to the KEGG (Kyoto Encyclopedia of Genes and Genomes) pathways and enrichment of various metabolic pathways are shown as a heatmap in Supplementary Fig. S5. According to cluster analysis, the three groups clustered together on d3 but were clearly separated on d7. Only seven metabolic pathways were significantly different among the three groups on d3 (Fig. 6A, P < 0.05). The activity of the seven metabolic pathways in the PM group was in notable contrast to that in the PT group. Compared to the PM and PT groups, the AF group showed an over-representation of the pentose phosphate pathway and a moderate representation of the remaining six pathways. On d7, 22 metabolic pathways exhibited significant differences among the three groups (Fig. 6B, P < 0.05). There was an almost opposite representation of the 22 pathways between the PM and AF group while the PT group displayed a relatively low presentation of these 22 pathways.

## Discussion

In the present study, we explored the changes in gut bacterial communities and metabolites in preterm neonates following one-week treatment with different combinations of β-lactam related antibiotics in comparison with antibiotic-free controls. The microbiota remained stable within the first three days following antibiotic treatment but changed significantly after one-week treatment, most notably with an increase in the composition of some pathogenic bacteria. Regardless of the antibiotic type, there was a decrease in the diversity of gut microbiota in both treatment groups (PM and PT groups) compared to the antibiotic-free control group. Meanwhile, the duration of antibiotic treatment also had significant effects on the development of gut metabolites and metabolic pathways. Significant changes were observed in the composition of gut metabolites as well as the representation of metabolic pathways only after one-week treatment.

It has been reported that a decrease in the diversity of gut microbiota could be detected at week one and week two after stopping a less than three days antibiotic treatment in infants, but at week three, the microbial diversity in these infants was restored to the same level as week one^11, 18^. These findings suggest that antibiotic treatment for a short duration may have only mild and temporary effects on the gut microbiota and their metabolites. In our study, we found that infants treated with penicillin-moxalactam had a less significant decrease in the diversity of gut microbiota compared to infants treated with piperacillin-tazobactam. A decreased microbiota diversity was pronounced among children with NEC and LOS^19, 20^, which are also common in preterm infants, and the decreased diversity may result from prolonged empiric antibiotic therapy. Therefore, a long-term antibiotic treatment may be a risk factor for serious infectious diseases, such as NEC and LOS.

In our study, Firmicutes and Proteobacteria were dominant in the intestinal microbiota of most samples, in agreement with previous studies on preterm babies^18^. Bacteroidetes, which is prominent in adults^21^, was very rare in our samples, supporting the notion that preterms often have a delayed colonization of normal anaerobic bacteria, including Bacteroidetes^3, 22^.

Both types of antibiotic treatment involved in this study led to overgrowth of stool *Streptococcus* in preterms. This observation is consistent with the general belief that antibiotics selectively kill sensitive bacteria and leave behind resistant ones that replicate and expand rapidly. *Streptococcus* is a genus of Gram-positive bacteria that can cause serious infections with high morbidity and mortality. *S*. *agalactiae*, for example, is the leading cause of neonatal sepsis. Although our study did not involve any infants with sepsis caused by *Streptococcus*, it has been reported that late-onset neonatal bloodstream infections can be caused by the enteric habitat of bacteria^23^, including *Streptococcus*, which usually colonizes in the mucosal layer of the intestinal tract and can spread to other organs causing serious infection^24^.

The administration of piperacillin-tazobactam in this study also resulted in overgrowth of stool *Enterococci*. Similar results have been reported in studies with NEC cases^25^. The *Enterococcus* is intrinsically resistant to several antibiotics and can cause nosocomial infections in patients who are debilitated by other concurrent illnesses or prolonged hospitalization. Previous studies, in an animal model with vancomycin-resistant enterococci (VRE) in stool, have found that treatment with anti-anaerobic antibiotics promoted high-density colonization^26^ and enabled exogenously administered VRE to efficiently displace the normal intestinal microbiota^27^. Several factors must be considered in evaluating the effects of antibiotics on intestinal microbiota, including the spectrum of antimicrobial activity and the level of active antibiotics in the intestinal tract. Some studies found that antibiotics promoted the overgrowth of VRE in the intestinal tract primarily through the inhibition of intestinal anaerobes^28^. This mechanism may explain the overwhelming intestinal colonization of *Enterococci* in infants in our study following treatment with the piperacillin-tazobactam combination, which is highly active against intestinal anaerobes though we did not explore antibiotic sensitivity patterns of the increased *Enterococci* organisms. The proportion of gram-positive bacteria significantly increased in infants after treatment with piperacillin-tazobactam in this study, which is consistent with the potent activity of this antibiotic against gram-negative bacilli.

In addition to the composition of gut microbiota, the metabolites of gut microbiota were also affected by antibiotic treatment in this study and this effect was dependent on the duration and type of the antibiotics used. The metabolites had no apparent changes within the first three days of treatment but showed significant changes after one-week treatment. Previous studies in rodents have demonstrated that broad-spectrum antibiotic therapy (including piperacillin-tazobactam) can disrupt the indigenous microbiota of the host, reduce the colonization resistance, and alter the intestinal metabolome, including both host- and microbial-derived metabolites^29, 30^. While these studies have shown that the changes of gut metabolites were a result of altered gut microbiota, it has not been determined whether the duration of antibiotic treatment affects the gut metabolites in preterm infants.

We found that following antibiotic treatment for 7 days, four fecal metabolites (linoleic acid, L-glutamate, L-valine, and pantothenic acid) increased significantly in AF group while two metabolites (L-tyrosine and citric acid) became dominant in PM group. Many metabolic pathways involving these six metabolites showed remarkable differences among the three groups. For example, the propanoate metabolism belongs to the carbohydrate metabolic pathways, including the malonate semialdehyde pathway and propanoyl-CoA pathway. It has been documented that propanoate metabolism is highly enriched simultaneously between the gut microbiota and the host^31^, and that the *Streptomyces*, which is the significant resource for antibiotic production, harbors the complete pathway of propanoate metabolism^32^. These studies suggest that propanoate metabolism plays an important role in the interaction between the host and its gut microbes and is necessary for normal metabolism. The disturbance of propanoate metabolism (deficiency of methylmalonyl-CoA mutase, propionyl-CoA carboxylase and malonyl-CoA decarboxylase) can cause many metabolic diseases characterized by developmental delay, seizure, hypoglycemia, cardiomyopathy and malonic aciduria. although the antibiotic treated preterm infants have no such clinical manifestations, the activity of propanoate metabolism was much lower in PT and PM groups than that in the antibiotic-free group on d7 indicating that the physiological metabolism is abnormal after antibiotic treatment in preterm infants.

We also observed that the penicillin and cephalosporin biosynthesis pathways were significantly different among the three groups on both d3 and d7 after birth. Penicillins are produced only by fungi, while cephalosporins (including cephamycins) are produced by fungi as well as bacteria. Both classes of antibiotics are synthesized from L-2-aminoadipate, L-cysteine and L-valine through a common pathway, which starts with the condensation of these three amino acids by the non-ribosomal peptide synthetase to form the tripeptide δ-(L-α-aminoadipyl)-L-cysteinyl-D-valine^33, 34^. In our study, the activity of the penicillin and cephalosporin biosynthesis pathways was higher in the AF group than that in the antibiotic treatment groups suggesting that the function of the penicillin and cephalosporin producer species decreased in the antibiotic treatment groups. These findings imply that the use of antibiotics can inhibit penicillin and cephalosporin biosynthesis and thus damage the natural defense barriers provided by the normal gut microbiota.

Our study has some limitations. First, both antibiotic treatment groups in this study were not sampled after day 7, and therefore the long-term effects of antibiotic treatments on gut microbiota could not be determined. Second, this study involves a small number of patients from the same hospital and the results may not be generalized to a larger population.

In summary, this study demonstrated profound effects of one-week antibiotic treatment on gut microbiota and their metabolites in preterm babies. A better understanding of the adverse effects of antibiotic therapy on gut microbiota could be translated into a better clinical practice in prescribing antibiotics to preterm infants.

## Materials and Methods

### Study subjects and sample collection

In this cohort, 36 newborn babies with a gestational age between 28 weeks and 37 weeks (referred as preterm infants) were enrolled shortly after birth from the Children’s Hospital of Chongqing Medical University, Chongqing, China. Infants with major congenital anomalies or malformations were excluded. All infants received enteral feedings of formula, without using any probiotics or prebiotics during the study period. The study was approved by the ethics commission of the Children’s Hospital of Chongqing Medical University and all protocols were carried out in accordance with the approved guidelines and regulations. Written informed consent was obtained from all parents of the infants involved in this study before sample collection.

The 36 enrolled babies were divided into three groups (12 babies per group), including the PM, PT and AF groups. Infants in the AF group were case-matched with infants in the PM and PT groups by gestational age (+/− one week), delivery mode, sex gender and birth weight.

Treatment regimens used include penicillin (10U kg-1, twice a day) combined with moxalactam (40 mg kg-1, twice a day) or piperacillin-tazobactam (75 mg kg-1, twice a day). Treatment started from the first postnatal day and continued for at least 7 days (prolonged antibiotics). The AF group was free of exposure to any antibiotics during the entire study period. All infants remained free of NEC, sepsis or death during the study period.

The meconium and fecal samples on d3 and d7 were collected by medical staff of the Department of Neonatology in the hospital. Freshly evacuated feces in diapers were collected into sterile tubes and transported to the laboratory immediately. All samples were stored at −80 °C until further processing.

### DNA extraction and PCR amplification

DNA was extracted from 250 mg fecal samples using the QIAamp FAST DNA Stool Mini-Kit (Qiagen, Germany) according to the manufacturer’s instructions. The DNA was eluted in a final volume of 100 μL ATE buffer supplied in the kit. The V3-V4 region of bacterial 16S rRNA gene was amplified by PCR using universal primers 338F, 5′-ACTCCTACGG-GAGGCAGCA-3′, and 806R, 5′-GGACTACHVGGGTWTCTAAT-3′, which contain an 8-base barcode sequence unique to each sample (not shown). PCR reactions were performed in a total volume of 20 μL including 10 ng of DNA template and 5 μM of each primer. Thermocycling conditions were 3 min at 95 °C, then 27 cycles of 30 s at 95 °C, 30 s at 55 °C and 45 s at 72 °C, with a final extension at 72 °C for 10 min.

### Illumina MiSeq sequencing

PCR amplicons were separated by 2% agarose gel electrophoresis, then purified using the AxyPrep DNA Gel Extraction Kit (Axygen Biosciences, U.S.) according to the manufacturer’s instructions. Following quantification using QuantiFluor™-ST (Promega, U.S.), amplicons were pooled in equimolar and paired-end sequenced (2 × 250) on an Illumina MiSeq platform according to the standard protocols. The raw reads were deposited into the NCBI Sequence Read Archive (SRA) database (with accession numbers SRP108340).

### Bioinformatic analysis of microbiota

Raw fastq files were demultiplexed, quality-filtered Using QIIME (version 1.17) according to the index sequence. Reads with ambiguous characters, more than one nucleotide mismatch in primer sequences, or a length shorter than 50 bp were removed. Reads, which could not be assembled, were also discarded. Operational Taxonomic Units (OTUs) were clustered at 97% similarity level using UPARSE version 7.1 (http://drive5.com/uparse/). The phylogenetic affiliation of each 16 S rRNA gene sequence was analyzed by RDP Classifier (http://rdp.cme.msu.edu/) against the silva 16 S rRNA database using a confidence threshold of 70%^35^. The Principal coordinates analysis (PCoA) was generated by using R package vegan 2.0 to demonstrate the clustering of different samples^36^. Venn diagrams were implemented by Venn Diagram, while Mantel test, Redundancy analysis (RDA), and Heatmap figures were performed in Vegan packages in R.

### Fecal metabolite extraction

Because three stool samples in PM group had insufficient volume, the microbiome analysis only included 9 stool samples in PM group on d3 and d7, respectively. Each baby of the PT and AF groups had one stool sample available on d3 and d7, respectively. For metabolite extraction, 100 mg fecal samples were transferred into 5- mL centrifuge tubes, and suspended in 500 μL ddH_2_O at 4 °C. Following mixing with 1,000uL of methanol (pre-cooled at −20 °C, the tubes were placed into an ultrasound machine at room temperature for 10 min, then stew for 30 min on the ice. The resulting solutions were centrifuged for 10 min at 14,000 rpm at 4 °C and 1.2 mL supernatant was collected into a new centrifuge tube, dried by vacuum concentration, and then dissolved in 400 μL methanol aqueous solution (1:1) at 4 °C. After filtering through 0.22 μm membrane, the final solution was subjected to quality control tests before liquid chromatography-mass spectrometry (LC-MS) analysis^37–39^ as described below.

### LC-MS-based metabolite profiling

Chromatographic separation was performed on an Acquity UPLC system equipped with an ACQUITY UPLC HSS T3 (150 × 2.1 mm, 1.8 µm, Waters) column maintained at 40 °C. An equilibrated sample (3 μL) was injected into the column. The electron spray ionization mass spectrometry (ESI-MS) experiments were executed on the Thermo LTQ-Orbitrap XL mass spectrometer with the spray voltage of 4.8 kV and 4.5 kV in positive and negative modes, respectively^37–39^.

### Bioinformatic analysis of metabonomics

Raw LC-MS data were converted into a mzXML file format and processed using the XCMS tool-box for automatic peaks detection, peaks filtration, and peaks alignment. The data were arranged in a data matrix consisting of mass to charge ratio (m/z), retention time and peak area. The XCMS output was further processed using Microsoft Excel for subsequent analysis.

### Statistical analysis

Statistical analysis was performed using SPSS version 22.0 for Windows (SPSS Inc., USA). If the data was normally distributed, they were expressed as mean ± SD; if not, expressed as the median and interquartile range (IQR). The differences among groups were analyzed using Fisher’s Exact test for categorical variables and Kruskal-Wallis Test for continuous variables after subsampling. Pairwise comparison was measured by White´s non-parametric t-test. A P value of <0.05 was considered statistically significant. Comparison of metabolites and metabolic pathways among the three groups was performed using one-way ANOVA, and the differences of metabolites were considered statistically significant with a P value of <0.05 + fold change ≥1.5 or ≤ 0.667^40, 41^.

## Electronic supplementary material

supplementary material
