# Supplementary material for: Effects of One-Week Empirical Antibiotic Therapy on the Early Development of Gut Microbiota and Metabolites in Preterm Infants
Source: Sci Rep. 2017 Aug 14;7:8025. doi: 10.1038/s41598-017-08530-9 (PMC5556106; doi:10.1038/s41598-017-08530-9)
Supplement: Supplementary file 1 — supplementary material [file 41598_2017_8530_MOESM1_ESM.pdf]

Effects of One-Week Empirical Antibiotic Therapy on the Early Development of Gut Microbiota and Metabolites in Preterm Infants

Danping Zhu[1,2,3,4,δ], Sa Xiao[1,2,3,4,δ], Jialin Yu[1,2,3,4,5], Qing Ai[1,2,3,4], Yu He[1,2,3,4], Chen Cheng[1,2,3,4], Yunhui Zhang[1,2,3,4], Yun Pan[1,2,3,4]

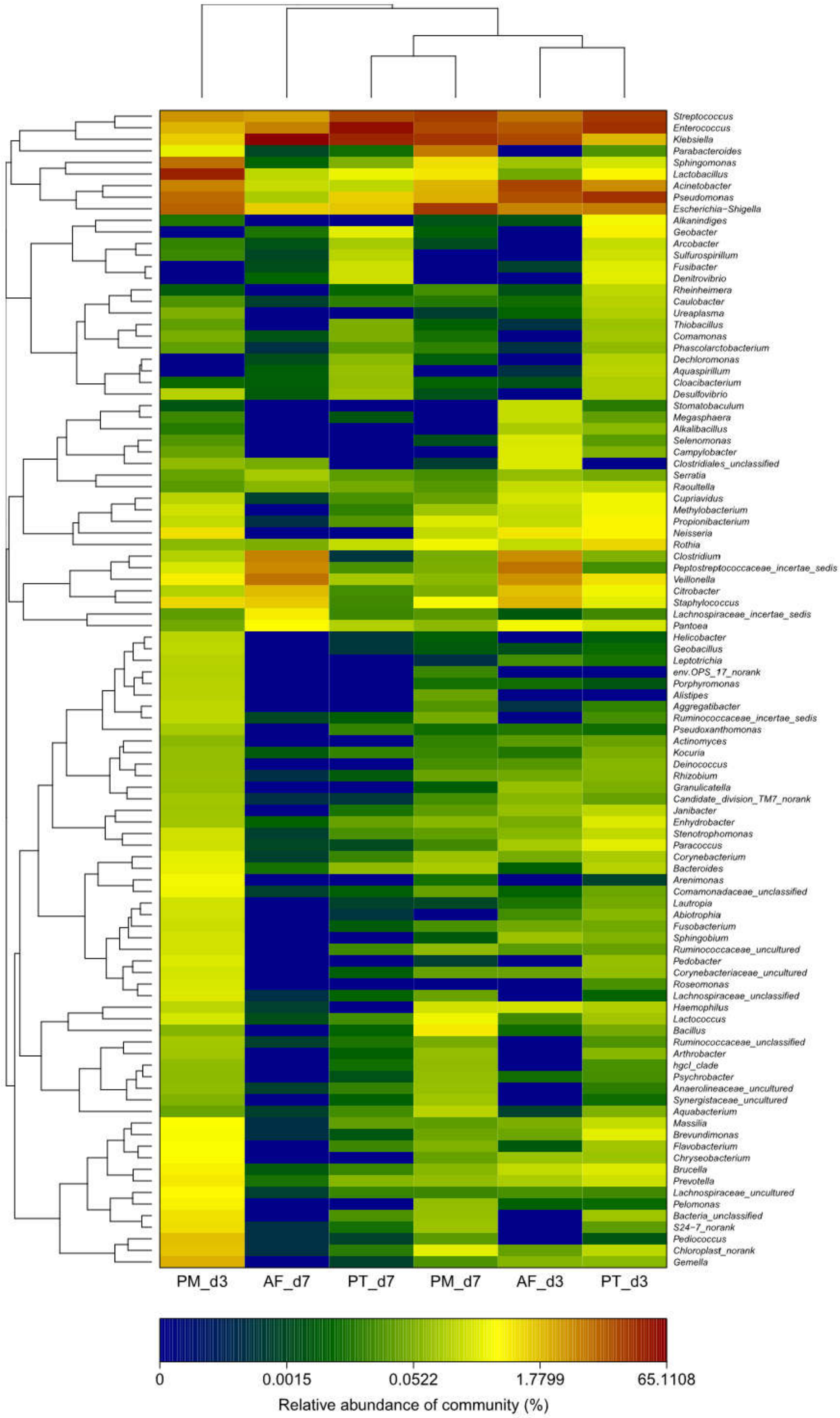

Supplementary Figure 1. Heatmap of microbial distribution of the top 100 abundant genera among all samples. AF, antibiotic-free group;

PT, piperacillin-tazobactam group; PM, combination of penicillin and moxalactam group; d3, day 3; d7, day 7.

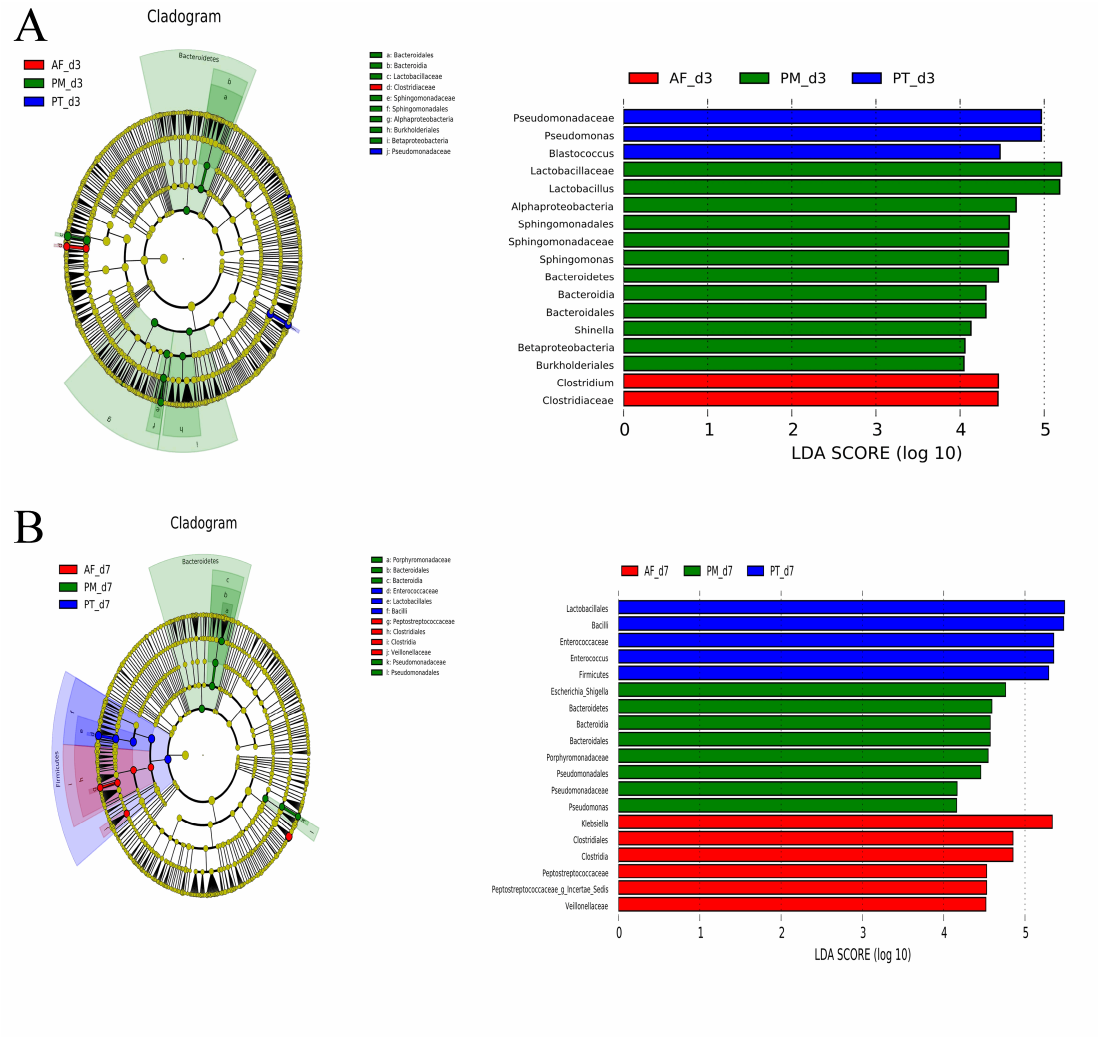

Supplementary Figure 2. LDA Effect Size (LEfSe) analysis of bacterial composition among the three study groups. LEfSe couples statistical significance with biological consistency and effect size estimation. The linear discriminant analysis (LDA) score represents the effect size and ranking of differentially abundant species. For the abbreviation of group names, see Supplementary Figure S1 legend.

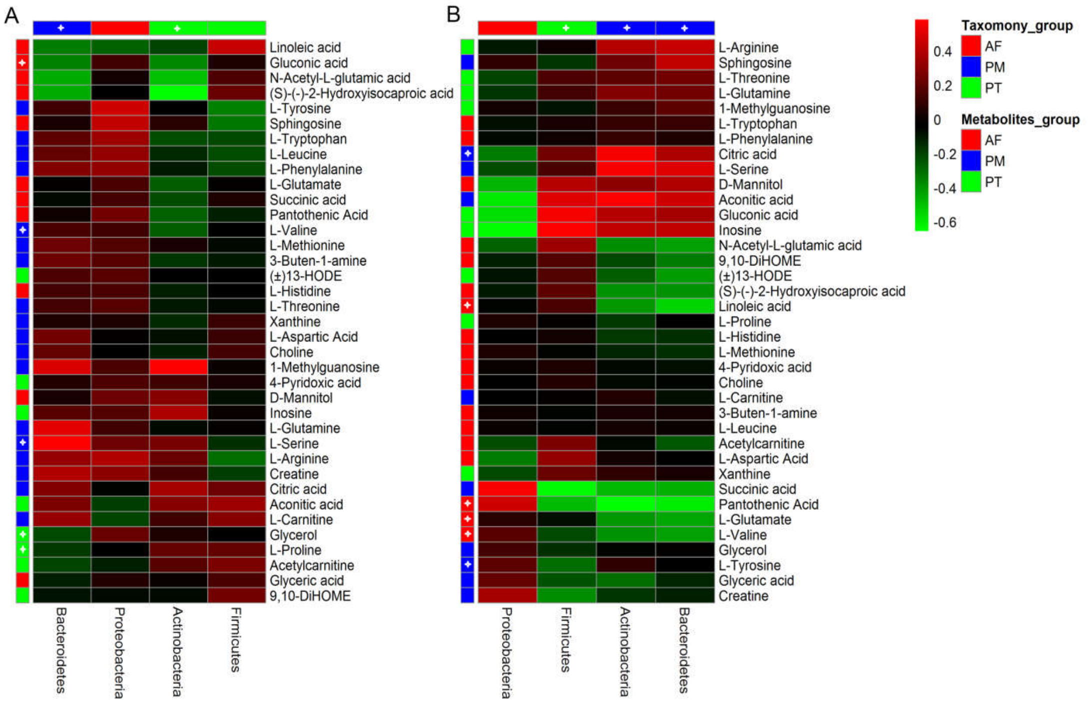

Supplementary Figure 3. Correlation analysis of all fecal metabolites and bacterial communities at the phylum level on day 3 (A) and day 7 (B). The correlation coefficient ranges from -0.6 to 0.4, with the red color representing positive correlation and the green color representing negative correlation. The heatmap refers to the main part containing 4 columns (with color changing from green to red) in the center of each panel. The horizontal bars on the top indicate the taxa of bacteria most abundant in each group (color-coded by the key). The vertical bars on the left side indicate the metabolites most abundant in each group (color-coded by key). The asterisk indicating the taxa of bacterium or metabolite that are significantly enriched among the three study groups ($P < 0.05$). For the abbreviation of group names, see Supplementary Figure S1 legend.

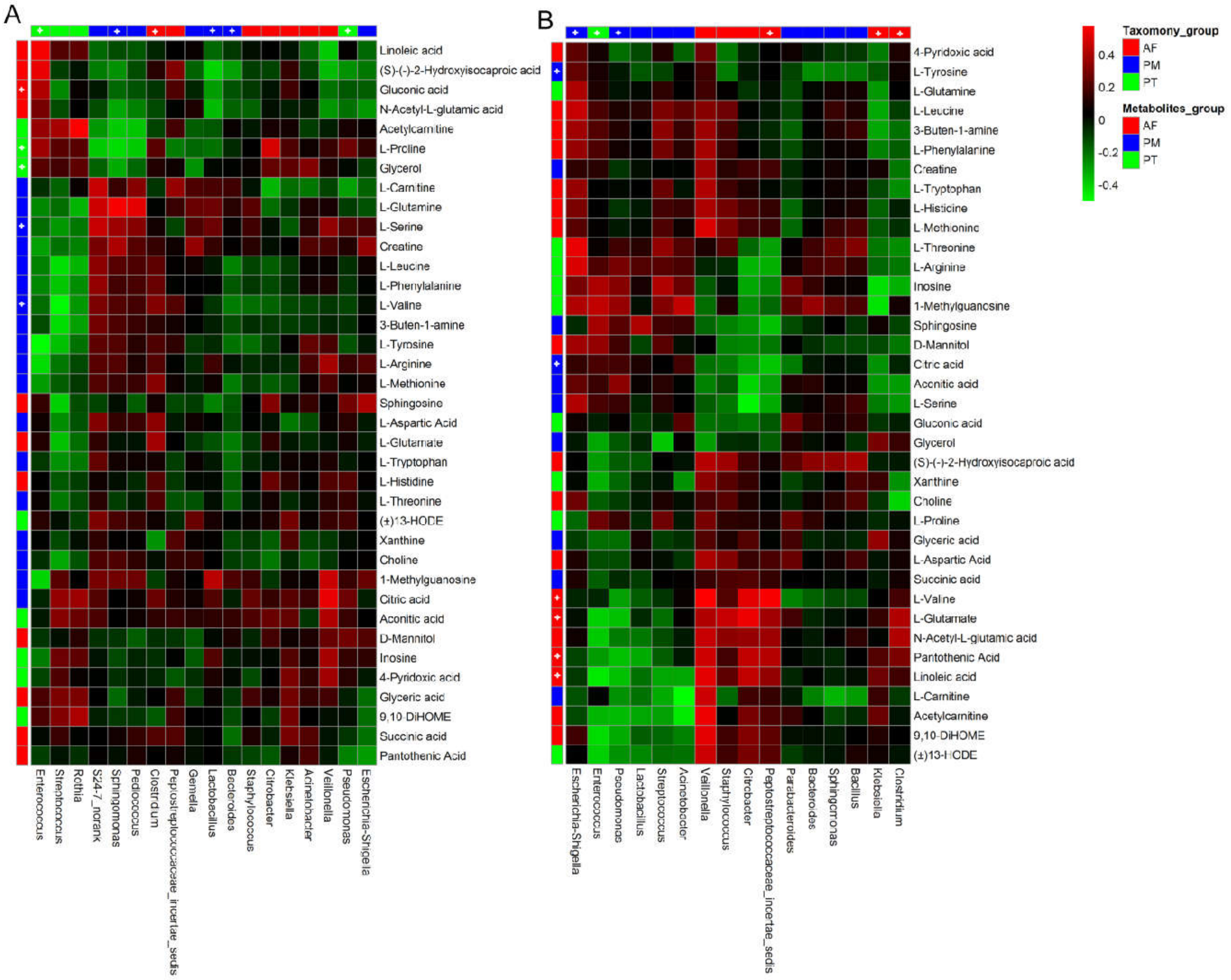

Supplementary Figure 4. Correlation analysis of fecal metabolites and bacterial communities at the genus level on day 3 (A) and day 7 (B). The correlation coefficient ranges from -0.4 to 0.4, with the red color representing positive correlation and the green color representing negative correlation. The heatmap refers to the main part containing columns (with color changing from green to red) in the center of each panel. The horizontal bars on the top indicate the taxa of bacteria most abundant in each group (color-coded by the key). The vertical bars on the left side indicate the metabolites most abundant in each group (color-coded by key). The asterisk indicating the taxa of bacterium or metabolite that are significantly enriched among the three study groups ($P < 0.05$). For the abbreviation of group names, see Supplementary Figure S1 legend.

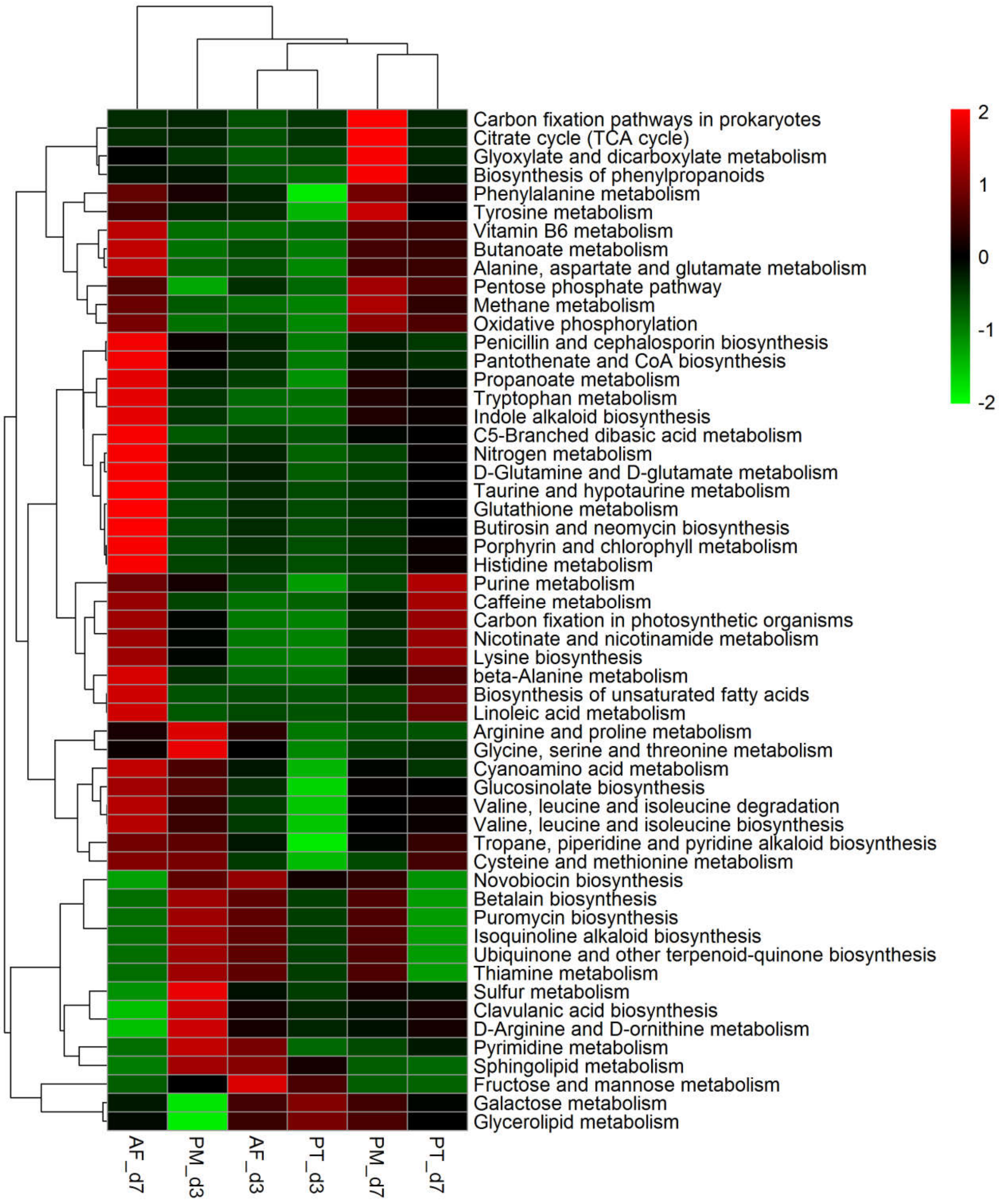

Supplementary Figure 5. Heatmap of metabolic pathways of the three study groups on day 3 and day 7. The activity score (AS) calculated by Pathway Activity Profiling (PAPi) ranges from -2 to 2. The cluster analysis was based on the processes of metabolism. For the abbreviation of group names, see Supplementary Figure S1 legend.
